# Case Report: Sequential Development of Three Mature Lymphoid Neoplasms in a Single Patient: Clonal Relationship and Molecular Insights

**DOI:** 10.3389/fonc.2022.917115

**Published:** 2022-06-06

**Authors:** Chiara Salvetti, Candida Vitale, Valentina Griggio, Daniela Drandi, Rebecca Jones, Lisa Bonello, Riccardo Bomben, Alberto Bragoni, Davide Bagnara, Franco Fais, Valter Gattei, Federica Cavallo, Alberto Zamò, Marta Coscia

**Affiliations:** ^1^ Division of Hematology, University of Torino, A.O.U. Città della Salute e della Scienza di Torino, Torino, Italy; ^2^ Department of Molecular Biotechnology and Health Sciences, University of Torino, Torino, Italy; ^3^ Molecular Pathology Unit, A.O.U. Città della Salute e della Scienza di Torino, Torino, Italy; ^4^ Clinical and Experimental Onco-Hematology Unit, Centro di Riferimento Oncologico, IRCCS, Aviano, Italy; ^5^ Pathology Unit, Department of Medical Sciences, University of Torino, Torino, Italy; ^6^ Department of Experimental Medicine, University of Genova, Genova, Italy; ^7^ U.O. Molecular Pathology, I.R.C.C.S. Policlinico San Martino, Genova, Italy; ^8^ Institute of Pathology, University of Würzburg, Würzburg, Germany

**Keywords:** Richter’s syndrome, chronic lymphocytic leukemia, monoclonal B-cell lymphocytosis, IGHV genes, Hodgkin lymphoma

## Abstract

Two main variants of Richter syndrome (RS) are recognized, namely, the diffuse large B-cell lymphoma (DLBCL) and the Hodgkin’s lymphoma (HL) variant. Clonal relationship, defined as an identity of the immunoglobulin heavy chain variable (IGHV) region sequence between chronic lymphocytic leukemia (CLL) and RS clones, characterizes patients with a poor prognosis. Due to method sensitivity, this categorization is performed without considering the possibility of small-size ancillary clones, sharing the same phenotype with the preexisting predominant CLL clone, but with different IGHV rearrangements. Here we describe and molecularly profile the peculiar case of a patient with a CLL-like monoclonal B-cell lymphocytosis (MBL), who sequentially developed a DLBCL, which occurred concomitantly to progression of MBL to CLL, and a subsequent HL. Based on standard IGHV clonality analysis, DLBCL was considered clonally unrelated to the concomitantly expanded CLL clone and treated as a *de novo* lymphoma, achieving a persistent response. Three years later, the patient further developed a clonally unrelated HL, refractory to bendamustine, which was successfully treated with brentuximab vedotin and radiotherapy, and later with pembrolizumab. We retrospectively performed additional molecular testing, by applying next-generation sequencing (NGS) of immunoglobulin repertoire (Ig-rep) techniques and a more sensitive allele-specific oligonucleotide-droplet digital PCR (ASO-ddPCR) strategy, in order to quantitatively investigate the presence of the rearranged IGHV genes in tumor specimens collected during the disease course. In this highly complex case, the application of modern and sensitive molecular technologies uncovered that DLBCL, initially considered as a *de novo* lymphoma, was instead the result of the transformation of a preexisting ancillary B-cell clone, which was already present at the time of first MBL diagnosis. A similar approach was also applied on the HL sample, showing its clonal unrelatedness to the previous MBL and DLBCL.

## Introduction

Richter syndrome (RS) is defined as the occurrence of an aggressive lymphoma in patients with a previous or concomitant diagnosis of chronic lymphocytic leukemia (CLL) (1). Approximately 2%–8% of patients with CLL may progress to RS, most frequently with a diffuse large B-cell lymphoma (DLBCL) variant, while the classical Hodgkin lymphoma (HL) variant is definitely less common (<10%) (2–4). Concomitant DLBCL and HL transformation has been reported in few cases (5, 6). The most relevant prognostic factor for RS is the clonal relationship between the preexisting CLL and the new-onset lymphoma: clonally related RS has poor prognosis, while clonally unrelated transformation usually behaves like *de novo* DLBCL, with a longer median survival (8–16 months vs. 5 years, respectively) (7). The HL variant is clonally unrelated in the majority of cases and has a relatively good prognosis, with a median survival of approximately 4 years (8, 9). The standard method to define clonal relationship is the study of the nucleotide sequence of the immunoglobulin heavy chain variable (IGHV) region rearrangement by polymerase chain reaction (PCR) and Sanger sequencing (SS). Clonally related RS is characterized by a nearly identical IGHV sequence in the secondary lymphoma and in the preexisting CLL clone (10). Notably, a clonal relationship with small-size MBL clones, sharing the same phenotype with a predominant clone, may be difficult to establish due to sensitivity issues with commonly used assays. Here we describe and molecularly characterize the peculiar case of a patient with a monoclonal B-cell lymphocytosis (MBL), who sequentially developed a DLBCL followed by a HL.

## Case Presentation

**Figure 1:**
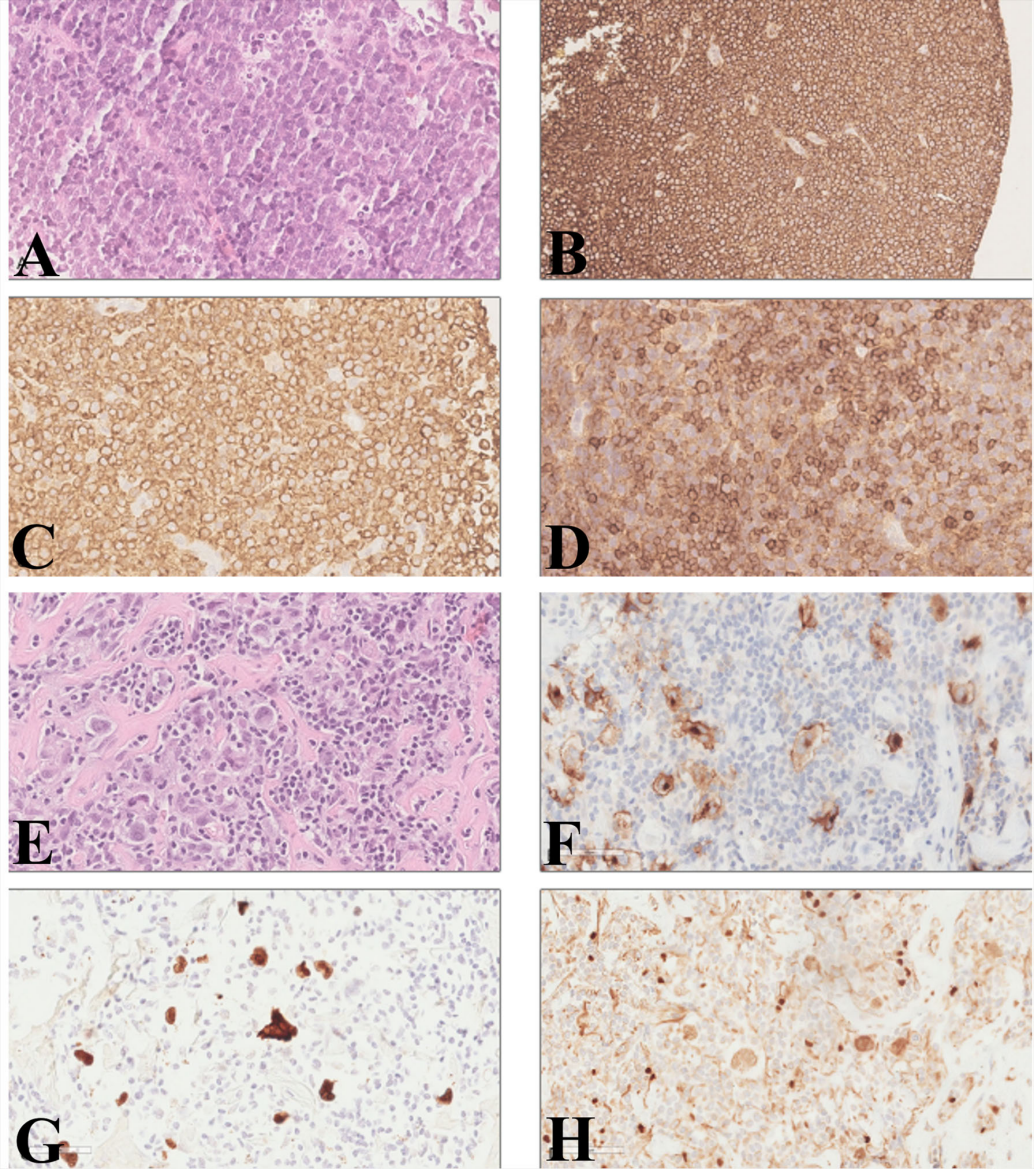
Diffuse large B-cell lymphoma and classical Hodgkin’s lymphoma diagnosis on lymph node biopsy. Large lymphoid cells with basophil cytoplasm, ovalar nuclei, and one or more nucleoli; small lymphocytes with a round nucleus were visible among the more numerous large cells (**A**, hematoxylin and eosin). The large cells were positive for CD20 **(B)** and BCL2 **(C)**. CD5 was strongly positive in the small cells, while the large cells were partially and weakly positive **(D)**. Large pleomorphic cells, with one or two ovalar nuclei and macro-nucleoli in a background of small lymphocytes and granulocytes with fibrosis (**E**, hematoxylin and eosin). The large cells were positive for CD30 **(F)** and PAX5 **(H)**. Immunohistochemical staining for EBV-encoded RNA (EBER) was positive **(G)**. (**A**, **C**, **D–H**: original magnification ×40; **B**: original magnifications ×20).

In 2010, a 64-year-old woman was diagnosed with CLL-like MBL. Nothing relevant was reported in the family history, and past medical history included only diverticulosis and a previous surgery for breast fibroadenoma. The patient presented a mild lymphocytosis (<5,000/µl) and normal ranges of hemoglobin and platelets, and the physical examination did not reveal any hepatomegaly, splenomegaly, or enlarged nodes. Immunophenotyping was performed and showed a B-cell clone accounting for 65% of circulating lymphocytes (corresponding to 3,900 monoclonal B cells/µl) expressing CD5, CD19, CD20, CD22, CD23, and CD43 and a weak expression of the surface immunoglobulin kappa chain (i.e., high-count CLL-like MBL). Concerning risk assessment, in 2010 a fluorescence *in situ* hybridization (FISH) performed on peripheral blood (PB) showed chromosome 13 deletion in 80% of the B-cell population and was negative for chromosome 11 and 17 deletion and for chromosome 12 trisomy. The patient was monitored over time, remaining stable for the following 3 years. In 2013, the patient showed evidence of rapid disease progression, with fever, fatigue, and increased lymphadenopathy. Laboratory tests showed an increase in lymphocytosis (24,420/µl), decreased platelets (64,000/µl), and markedly elevated LDH level (1,217 IU/l). A flow cytometric analysis demonstrated a monoclonal B-cell population with the same phenotypical markers of the initial MBL, including kappa light chain restriction and expression of both CD5 and CD23; FISH analysis confirmed chromosome 13 deletion in 98% of B cells. A PET/CT scan showed numerous lymph nodes up to 5 cm on both sides of the diaphragm and skeletal involvement in multiple sites, with high standardized uptake value (SUV). Due to the rapid deterioration of clinical conditions, a biopsy of the bone marrow and of a fast-growing superficial lymph node described in the PET/CT scan was quickly performed, for diagnostic purposes. The bone marrow biopsy—only partially informative because of the presence of extensive necrosis—showed the presence of large lymphoid cells, CD20+, CD5+, and CD79a+. The pathology report of the lymph node biopsy described few small lymphoid cells consistent with CLL, surrounded by an extensive infiltration of large lymphoid cells. The large cells were positive for CD20 and BCL2. CD5 was strongly positive in the small cells, while the large cells were partially and weakly positive. The Ki-67 proliferation index was 70% for the large cells and 5% for the small cells. The pathologist concluded for a DLBCL (**Figure 1**). The assessment of Epstein–Barr virus (EBV) presence by immunohistochemistry was not feasible due to insufficient lymph node and bone marrow material. Based on clinical and radiological evaluations, the DLBCL was classified as stage IVB based on Lugano classification (11). Direct sequencing of immunoglobulin gene rearrangement performed by SS on the lymph node biopsy showed an IGHV clonal rearrangement (clone “R”: IGHV3-11*01, with a 96% homology to the germline), which was different from the clone detected in the PB sample collected at the time of MBL diagnosis (clone “D”: IGHV3-23*02, with an 89.7% homology to the germline). Interestingly, neither clone “D” (i.e., the first clone identified in the PB at the time of MBL diagnosis) nor clone “R” (i.e., the clone identified in the lymph node biopsy at the time of DLBCL) carried a CLL stereotyped B-cell receptor (10). The patient was treated with six cycles of rituximab, cyclophosphamide, vincristine, liposomal doxorubicin, and prednisone (R-COMP) followed by two additional infusions of rituximab and two cycles of intravenous methotrexate, as central nervous system prophylaxis. Therapy was well tolerated, and the patient achieved a complete response with a normalization of the blood count (absolute lymphocyte count, 1,130/µl) and a post treatment bone marrow biopsy negative for DLBCL or CLL infiltration.

The patient subsequently underwent clinical and radiological follow-up. In 2016, due to the appearance of a right leg edema, a CT scan was performed and noted the presence of iliac and inguinal lymphadenopathies up to 6.5 cm, with an SUV of 15 at PET scan. A lymph node biopsy was performed, showing large Reed–Sternberg cells in a background of small lymphocytes and granulocytes with fibrosis. The large cells were positive for CD30 and PAX5. As already reported in more than half of HLs arising from CLL (12), immunohistochemical staining for EBV-encoding RNA (EBER) was positive and the pathologist concluded for EBV-positive, nodular sclerosis classical HL (cHL) (stage IIA) (**Figure 1**). As often reported in HL-RS (8), clonality evaluation performed by multiplex PCR followed by GeneScan analysis (13) showed a polyclonal result. The bone marrow biopsy was negative for HL or DLBCL involvement, showing only 1.2% of CLL cells by immunophenotypic analysis. The patient was initially treated with six cycles of bendamustine, but restaging PET and CT scans showed a progressive disease with new abdominal lesions. Therefore, the patient received eight cycles of immunotherapy with anti-CD30 antibody–drug conjugate brentuximab vedotin, thus achieving a complete response in April 2017. In August 2019, a histological examination confirmed HL recurrence, involving inguinal, iliac, and obturator lymph nodes, in the absence of clinical symptoms (stage IIA). The patient therefore received four additional cycles of brentuximab vedotin followed by radiotherapy (30 Gy), thus achieving again a complete response. In 2021, the CT scan detected a new disease relapse, as shown by the presence of iliac and abdominal lymphadenopathies up to 5.3 and 2.6 cm, respectively, with high SUV at the PET scan. The patient was initiated on treatment with pembrolizumab 200 mg q3 weeks, thus achieving a complete response that is persisting after a total of 15 administrations, at the time of writing this report.

**Figure 2:**
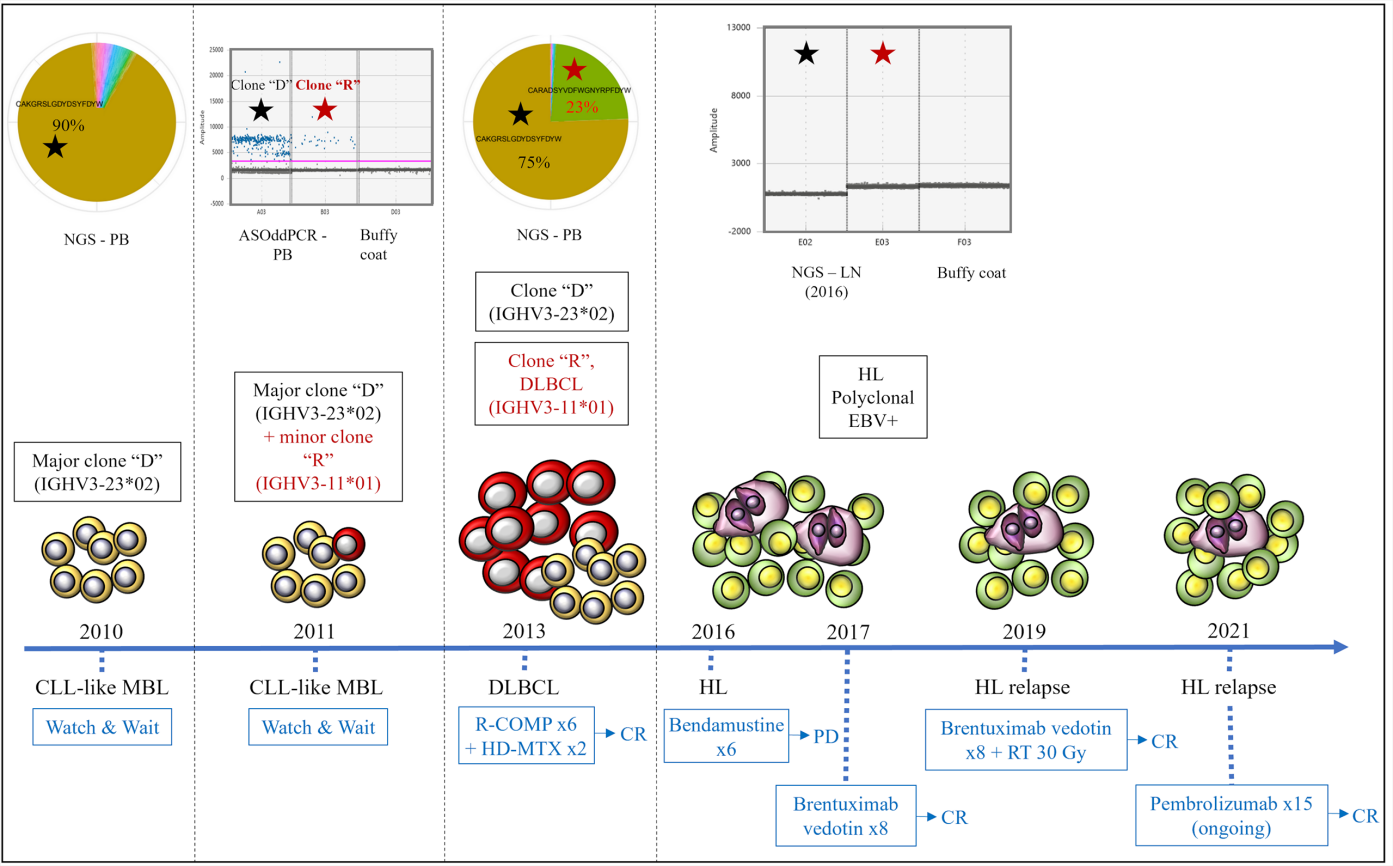
Clonal relationships by Ig-rep and ASO-ddPCR and clinical evolution of the three lymphoid neoplasms. Clone “D” (indicated with a black star) was detected in the PB sample collected at the time of MBL diagnosis and was persistent in the PB collected at the time of DLBCL. Clone “R” (i.e., the clone identified in the lymph node biopsy at the time of DLBCL transformation, indicated with a red star) was undetectable by NGS of Ig-rep in the PB sample collected at the time of MBL diagnosis, whereas it became detectable by ASO-ddPCR in an interim PB sample collected during the “watch & wait” period (where it coexisted with clone “D”). Neither clone “D” nor clone “R” were detectable in the lymph node specimen collected at the time of HL. ASO-ddPCR, allele-specific oligonucleotide-droplet digital PCR; NGS, next-generation sequencing; Ig-rep, immunoglobulin repertoire; PB, peripheral blood; LN, lymph node; DLBCL, diffuse large B-cell lymphoma; HL, Hodgkin’s lymphoma; MBL, monoclonal B-cell lymphocytosis; EBV, Epstein–Barr virus; R-COMP, rituximab, cyclophosphamide, liposomal doxorubicin, vincristine, prednisone; HD-MTX, high-dose methotrexate; CR, complete response; PD, progressive disease; RT, radiotherapy.

Based on the peculiarity of the case, we retrospectively performed additional molecular tests applying next-generation sequencing (NGS) of immunoglobulin repertoire (Ig-rep) techniques and a more sensitive allele-specific oligonucleotide-droplet digital PCR (ASO-ddPCR) strategy to quantitatively investigate the presence of the rearranged IGHV genes in tumor specimens collected at different timepoints during the disease course (14, 15). The Ig-rep was performed from gDNA with VH-FR1 BIOMED2 primers (16) and sequenced with Illumina MiSeq 2x300 cycles. Raw sequences were analyzed as described in Vergani et al. (17). For ASO-ddPCR IgH, rearranged variable regions (VDJ) were directly sequenced from an MBL PB sample and from a DLBCL lymph node sample, and the complementarity-determining regions (CDR) were identified and used to generate tumor-specific (ASO) primers, although accounting for somatic hypermutations that can affect the primer-binding sites (14, 18). The burden of clone “D” was persistent in the PB and in the lymph node collected at the time of RS. By contrast, clone “R” was i) undetectable in the PB sample collected at the time of MBL diagnosis, by both Ig-rep (**Figure 2**) and ASO-ddPCR (data not shown); ii) detectable by ASO-ddPCR—but not by standard SS (data not shown)—in an interim PB sample collected during the “watch & wait” period (i.e., 1 year after the first finding of MBL, in 2011), where it coexisted with the more represented clone “D” (**Figure 2**); and iii) detectable, as well as clone “D,” in the PB collected at the onset of DLBCL (2013), as shown by both Ig-rep (**Figure 2**) and ASO-ddPCR (data not shown). A similar ASO-ddPCR-based approach was also applied on the HL sample, showing that neither clone “D” nor clone “R” was detectable in the collected lymph node specimen, thus suggesting a clonal unrelatedness with the two previous MBL and DLBCL.

Our experience with this unusual case suggests that a reasonable approach could include Ig-rep at clinically relevant timepoints (such as diagnosis and disease transformation), while a highly sensitive, but simpler and less expensive, clone-specific ASO-ddPCR could be retrospectively applied to follow the appearance and progress of one or more specific clones during the patient’s history.

We also analyzed the mutational profiles of MBL and DLBCL using an amplicon-based NGS for 14 selected genes, which are known to be recurrently mutated in B-cell lymphomas (*TP53*, *NOTCH1*, *BTK*, *PLCG1*, *NFKBIE*, *MYD88 L265P*, *MAP2K1*, *MAP1K1*, *ASXL1*, *POT*, *KRAS*, *NRAS*, *BRAF*, *FBXW7*), as previously reported (15). The analysis was performed comparing paired PB samples obtained at MBL diagnosis and at DLBCL occurrence and resulted negative for all the analyzed mutations.

## Discussion

We describe the sequential occurrence of a DLBCL and cHL in a patient initially diagnosed with a CD5+ MBL. At the moment of the DLBCL diagnosis, the MBL had progressed to CLL, as demonstrated by the increased lymphocytosis and the immunophenotype on PB. Based on standard sequencing of Ig gene rearrangement performed by SS, the DLBCL was considered as a *de novo* lymphoma, clonally unrelated to the coexisting CLL, and was treated accordingly. Notably, the combination between Ig-rep and ddPCR molecular analyses showed that the DLBCL was derived from the transformation of a minor CD5+ ancillary clone that was already detectable during the MBL phase. Our observations are in line with previous data, suggesting that i) MBL cases often contain multiple B-cell clones (19) and ii) a proportion of DLBCL may arise from small precursor cells, with either a CLL-like or a non-CLL-like immunophenotype (20, 21). In the present case, DLBCL occurred in the absence of known risk factors predisposing to RS transformation, such as previous administration of multiple lines of treatment or the presence of genetic predisposing factors, such as *TP53* and *NOTCH* abnormalities or complex karyotype (22, 23). In addition, the good clinical outcome of DLBCL was more in line with a *de novo* lymphoma than with a clonally related RS and supported the notion that the biologic mechanisms underlying evolution from CLL-like MBL to overt aggressive lymphoma may be very different from those leading to RS transformation from advanced-stage or previously treated CLL. During the post treatment remission phase of the DLBCL, the patient was diagnosed with a further lymphoid neoplasm consisting in a secondary and probably EBV-driven cHL, likely unrelated to the previous MBL clone and displaying a favorable outcome after treatment with the anti-CD30 antibody–drug conjugate brentuximab vedotin. This case presents a high degree of complexity, at least partially linked to the presence of a heterogeneous tumor, consisting of three different and sequentially occurring mature lymphoid neoplasms (i.e., MBL, DLBCL with concomitant CLL and cHL)—thus defined according to the 2016 revision of the World Health Organization classification of lymphoid neoplasms (24). CLL is a B-cell malignancy characterized by several immune dysfunctions associated with increased risk of developing second malignancies, including additional clonally unrelated B-cell neoplasms (25, 26). Therefore, we may speculate that in this patient the development of three mature lymphoid neoplasms might have been at least favored by extrinsic microenvironmental mechanisms, such as an impaired immunological surveillance, which facilitate the expansion and progression of different neoplastic clones, rather than being the consequence of high-risk intrinsic genetic alterations, which generally confer a more pronounced clinical aggressiveness and therapy resistance.

## Author Contributions

CS, CV, and MC performed the diagnosis and follow-up, developed the idea, analyzed the data, and wrote the manuscript. DD, VGa, DB, RB, and FF performed the molecular tests and analyzed the data. LB, AB, and AZ performed the histologic analysis. FC performed the follow-up and participated in editing the manuscript. RJ, RB, and VGr analyzed the data and participated in editing the manuscript. All authors contributed to the article and approved the submitted version.

## Funding

DB has funding from the European Union’s Horizon 2020 research and innovation program under the Marie Skłodowska-Curie grant agreement no. 794075.

## Conflict of Interest

The authors declare that the research was conducted in the absence of any commercial or financial relationships that could be construed as a potential conflict of interest.

## Publisher’s Note

All claims expressed in this article are solely those of the authors and do not necessarily represent those of their affiliated organizations, or those of the publisher, the editors and the reviewers. Any product that may be evaluated in this article, or claim that may be made by its manufacturer, is not guaranteed or endorsed by the publisher.
